# Novel expression cassettes for increasing apolipoprotein AI transgene expression in vascular endothelial cells

**DOI:** 10.1038/s41598-022-25333-9

**Published:** 2022-12-06

**Authors:** Meena Sethuraman, Nagadhara Dronadula, Lianxiang Bi, Bradley K. Wacker, Ethan Knight, Pieter De Bleser, David A. Dichek

**Affiliations:** 1 Department of Medicine, University of Washington, Seattle, WA USA; 2 Department of Biomedical Molecular Biology, Ghent University, Ghent, Belgium; 3 Data Mining and Modeling for Biomedicine, VIB Center for Inflammation Research, Ghent, Belgium

**Keywords:** Cardiology, Molecular medicine, Molecular biology, Transcription

## Abstract

Transduction of endothelial cells (EC) with a vector that expresses apolipoprotein A-I (*APOAI*) reduces atherosclerosis in arteries of fat-fed rabbits. However, the effects on atherosclerosis are partial and might be enhanced if *APOAI* expression could be increased. With a goal of developing an expression cassette that generates higher levels of *APOAI* mRNA in EC, we tested 4 strategies, largely in vitro: addition of 2 types of enhancers, addition of computationally identified EC-specific cis-regulatory modules (CRM), and insertion of the rabbit *APOAI* gene at the transcription start site (TSS) of sequences cloned from genes that are highly expressed in cultured EC. Addition of a shear stress-responsive enhancer did not increase *APOAI* expression. Addition of 2 copies of a *Mef2c* enhancer increased *APOAI* expression from a moderately active promoter/enhancer but decreased *APOAI* expression from a highly active promoter/enhancer. Of the 11 CRMs, 3 increased *APOAI* expression from a moderately active promoter (2–7-fold; *P* < 0.05); none increased expression from a highly active promoter/enhancer. Insertion of the *APOAI* gene into the TSS of highly expressed EC genes did not increase expression above levels obtained with a moderately active promoter/enhancer. New strategies are needed to further increase *APOAI* transgene expression in EC.

## Introduction

Vascular endothelial cells (EC) have several important physiological roles including modulation of vascular tone and permeability, control of hemostasis, and regulation of leukocyte trafficking during inflammatory reactions^1^. EC are easily accessible via intravenous or intraarterial injection of gene-transfer vectors and therefore have been targets for gene-therapy approaches that aim to treat both local and systemic diseases^2–4^. EC are particularly attractive targets for gene therapy that prevents or treats atherosclerosis because they regulate entry of both inflammatory cells and cholesterol (major components of atherosclerotic lesions) into the blood vessel wall^5,6^.

Effective atheroprotective gene therapy, whether delivered via EC or other cells, requires expression cassettes that achieve high-level transgene expression^7^. In earlier work developing EC-targeted gene therapy for atherosclerosis, we showed that EC overexpression of apolipoprotein AI (*APOAI*)—using an expression cassette containing the cytomegalovirus (CMV) immediate early promoter—can slow or reverse atherosclerosis in hyperlipidemic rabbits^8–10^. However, these protective effects were partial and *APOAI* transgene expression declined over time^9,10^. We considered that EC-targeted atheroprotective gene therapy with *APOAI* might be enhanced through use of expression cassettes that express higher and more stable levels of *APOAI*, optimally with EC specificity to avoid ectopic transgene expression. Use of a more potent expression cassette might also allow use of lower vector doses that minimize vector-related toxicity^11,12^.

To pursue this goal, several years ago we generated a potent EC-specific expression cassette containing a modified mouse endothelin-1 (*Edn1*) enhancer-promoter (termed 4XETE) and a posttranslational regulatory element (together termed the “4XETE-oPRE” cassette)^13,14^. This cassette had relative EC-specificity and expressed an interleukin-10 transgene at levels at least as high as the CMV promoter in EC both in vitro and in vivo, with no decline in interleukin-10 transgene expression over time^14,15^. We then tested whether the 4XETE-oPRE cassette could also express *APOAI* at similarly high and stable levels. Remarkably, in vitro expression of *APOAI* from the 4XETE-oPRE cassette exceeded levels achieved with the CMV promoter; however, in vivo expression from the 4XETE-oPRE cassette was initially lower than from the CMV cassette^15^.

To further increase *APOAI* expression above levels achieved with the CMV or the 4XETE-oPRE cassettes in vivo (with the expectation that higher *APOAI* levels would lead to greater atheroprotection), we next tested several strategies including codon optimization of the *APOAI* transgene, complete removal of the 3′ untranslated region of *APOAI*, and co-expression of interleukin-10 with *APOAI* (to blunt local immune responses). However, none of these strategies increased *APOAI* expression^15^. Here we continued to pursue our goal of generating an expression cassette that increases EC *APOAI* expression above levels achieved with the CMV or 4XETE-oPRE-containing cassettes by testing several new strategies: addition of cell-type-specific enhancer sequences reported to be active in EC; addition of endothelial-specific cis-regulatory modules (CRM; effective in increasing transgene expression in other cell types, in some cases by > 100-fold)^16–18^; and insertion of the *APOAI* gene at the transcription start site of genomic sequences cloned from 4 genes that are highly expressed in cultured EC.

## Results

### Addition of a shear stress response element (SSRE) does not increase *APOAI* expression

**Figure 1:**
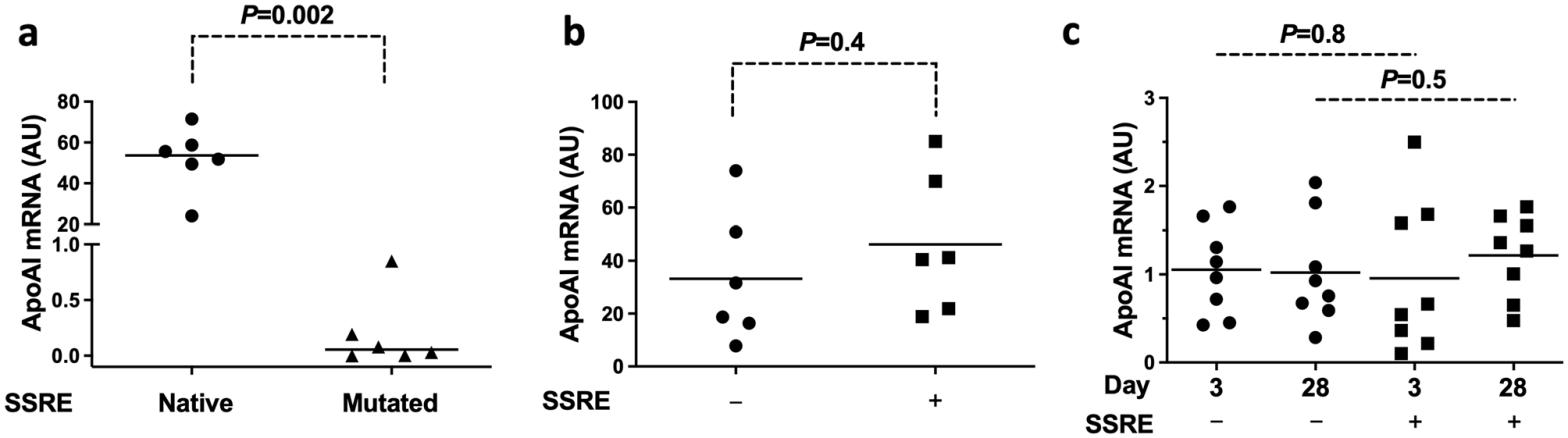
Placement of a shear stress response element (SSRE) upstream of the 4XETE-gApoAI-oPRE expression cassette. (**a**) Bovine aortic endothelial cells (BAEC) were transfected either with a plasmid with the 4XETE-gApoAI-oPRE cassette (including a native SSRE sequence on the “noncoding” stand) or a plasmid in which this SSRE was mutated. (**b**) BAEC were transfected with plasmids either without (−) or with (+) an exogenous SSRE. (**c**) Rabbit carotid endothelial cells were transduced in vivo with HDAd vectors including the 4XETE-gApoAI-oPRE expression cassette either without (−) or with (+) an exogenous SSRE. Carotid arteries were removed on day 3 or 28 after vector infusion and *APOAI* mRNA was measured by RT-qPCR. (**a**,**b**) *APOAI* mRNA was measured by RT-qPCR, 24 h after transfection. Data are from three independent experiments (**a**) or two independent experiments (**b**). Bars are medians (**a**) and means (**b**). (**c**) Data points are individual arteries; bars are means.

To test the activity of an exogenous SSRE placed upstream of our current highest-expressing *APOAI* cassette (4XETE-gApoAI-oPRE^14,15^; Supplementary Fig. S1), we first mutated an endogenous SSRE sequence (5′-GAGACC-3′)^19^ that we identified in the “noncoding” strand of the murine *Edn1* promoter (the *Edn1* promoter is included in the 4XETE sequence). Mutation of this sequence to 5′-ATGTCA-3′ (in the 4XETE-gApoAI-oPRE-mSSRE vector; Supplementary Fig. S1) resulted in near-complete loss of *APOAI* expression in bovine aortic endothelial cells (BAEC) under static conditions (> 99%; *P* = 0.002; Fig. 1a). Because this sequence seemed essential to the activity of the 4XETE promoter/enhancer even under static conditions, we left it intact in vectors designed to test whether addition of an exogenous SSRE could increase shear-responsive expression from the 4XETE-gApoAI-oPRE cassette.

We next transfected BAEC with plasmids containing the 4XETE-gApoAI-oPRE and SSRE-4XETE-gApoAI-oPRE expression cassettes (Supplementary Fig. S1). The latter plasmid has an exogenous SSRE (5′-GAGACC-3′)^19^ placed upstream of the 4XETE sequence but is otherwise identical to the plasmid containing 4XETE-gApoAI-oPRE. Addition of the SSRE had no effect on *APOAI* expression in BAEC maintained under static conditions (Fig. 1b; *P* = 0.4). To test if this SSRE increases *APOAI* expression in response to fluid shear stress in vivo, we transferred the SSRE-4XETE-gApoAI-oPRE expression cassette into an HDAd vector and compared it to HDAd-4XETE-gApoAI-oPRE in vivo in rabbit carotid arteries, using a model of EC-specific in vivo gene transfer^20,21^. Addition of the SSRE to 4XETE-gApoAI-oPRE did not alter APOAI expression (Fig. 1c; *P* = 0.8 and 0.5 at 3 and 28 days, respectively).

### Addition of a *Mef2c* enhancer element increases expression from a moderately active promoter/enhancer but not from a highly active promoter/enhancer

**Figure 2:**
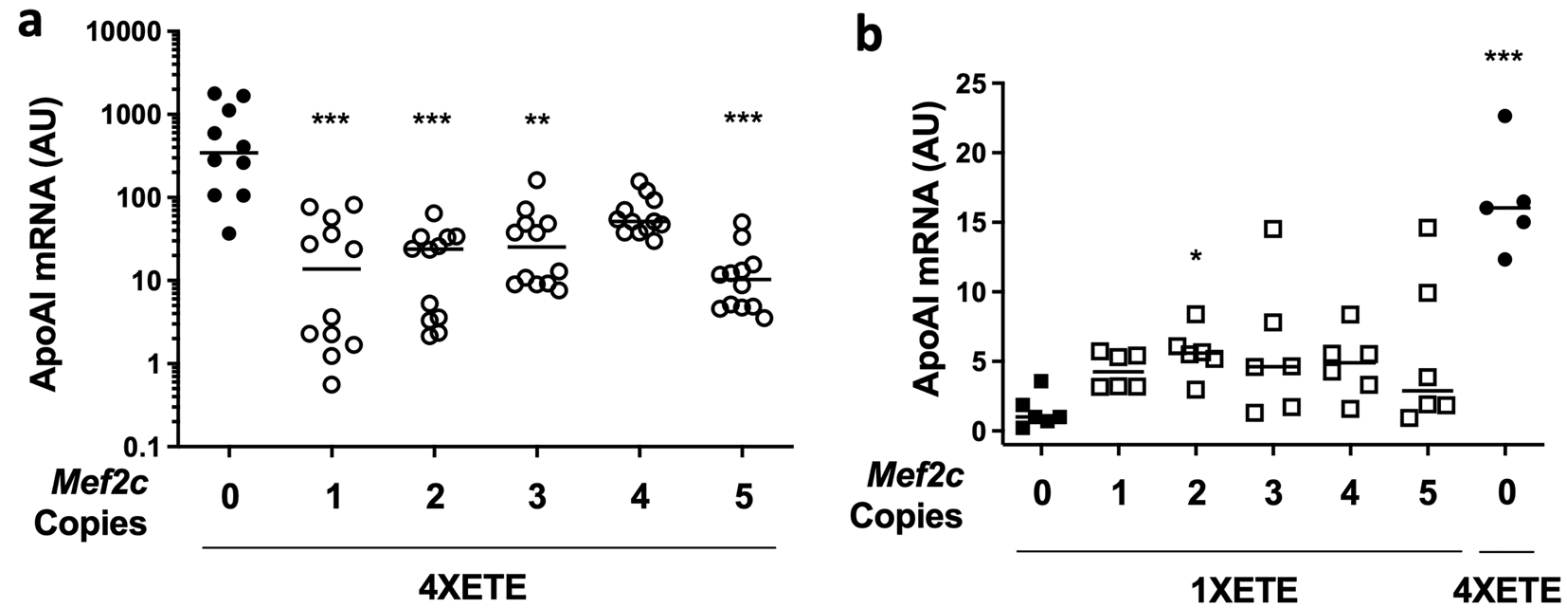
Addition of *Mef2c* enhancers to *APOAI* expression cassettes. (**a**) Bovine aortic endothelial cells (BAEC) were transfected with pBshuttle-4XETE-gApoAI-oPRE containing 0–5 copies of a *Mef2c* enhancer. (**b**) BAEC were transfected with pBshuttle-1XETE-gApoAI-oPRE containing 0–5 copies of a *Mef2c* enhancer, or with pBshuttle-4XETE-gApoAI-oPRE. (**a**,**b**) *APOAI* mRNA was measured by RT-qPCR, 24 h after transfection, and is expressed in arbitrary units (AU). (**b**) Median *APOAI* expression for 1XETE-gApoAI-oPRE with 0 *Mef2c* copies is assigned a value of 1; all other points are expressed relative to this value. (**a**,**b**) Data are from three independent experiments; bars indicate group medians. (**a**) **P < 0.01; ***P < 0.001 vs. 4XETE-gApoAI-oPRE with 0 *Mef2c* copies. (**b**) *P < 0.05; ***P < 0.001 vs. 1XETE-gApoAI-oPRE with 0 *Mef2c* copies.

We next tested the activity of a 44-bp enhancer element in the mouse *Mef2c* locus. When placed upstream of a heterologous promoter, this sequence is sufficient to direct vascular EC-specific expression in zebrafish embryos^22^. This *Mef2c* enhancer is highly evolutionarily conserved, and is bound/activated by Fox and Ets transcription factors. Similar FOX:ETS motifs are present in several EC-specific enhancers^22^, suggesting that placement of *Mef2c* enhancer copies upstream of the 4XETE promoter/enhancer could recruit EC-expressed transcription factors and increase *APOAI* expression. We tested whether addition of this *Mef2c* enhancer could increase expression from our highest-expressing cassette (4XETE-gApoAI-oPRE)^14,15^, by inserting 1–5 copies of the *Mef2c* enhancer upstream of the 4XETE promoter/enhancer in pBshuttle-4XETE-gApoAI-oPRE (Supplementary Fig. S1). Transfection of these constructs into BAEC (Fig. 2a) showed that addition of *Mef2c* enhancers either decreased *APOAI* expression (1, 2, 3 and 5 enhancer copies; *P* < 0.01 for all) or had no significant effect (4 copies; *P* = 0.6).

We considered that competition for transcription-factor binding between the *Mef2c* enhancer and the 4 *Edn1* enhancers in 4XETE might account for failure of the *Mef2c* enhancer to increase *APOAI* expression above levels obtained with 4XETE-gApoAI-oPRE. We also considered a related observation: that some enhancers activate transcription more potently when they are adjacent to a moderately active promoter than when they are adjacent to a highly active promoter (e.g., the moderately active *Palm* promoter versus the highly active *TTR* promoter, both tested in hepatocytes)^16^. Accordingly, we inserted 1–5 copies of the *Mef2c* enhancer upstream of the moderately active 1XETE promoter in pBshuttle-1XETE-gApoAI-oPRE (Supplementary Fig. S1; the 1XETE promoter, with only 1 copy of the *Edn1* enhancer, expresses *APOAI* at far lower levels than 4XETE)^14^. Transfection of BAEC with these constructs revealed that the construct that included 2 copies of the *Mef2c* enhancer upstream of 1XETE-gApoAI-oPRE (2XMEF2C-1XETE-gApoAI-oPRE) significantly increased *APOAI* expression, compared to the 1XETE-gApoAI-oPRE cassette (5.6-fold; *P* < 0.05; Fig. 2b). In contrast, *APOAI* expression from constructs containing 1, 3, 4, or 5 copies of the *Mef2c* enhancer did not differ significantly from the 1XETE-gApoAI-oPRE cassette. *APOAI* expression from the 4XETE-gApoAI-oPRE construct (included as a positive control) was higher than from 2XMEF2C-1XETE-gApoAI-oPRE (16-fold above 1XETE-gApoAI-oPRE; *P* < 0.001; Fig. 2b).

### Identification of EC-specific CRM and testing in BAEC, HAEC, and HUVEC

**Figure 3:**
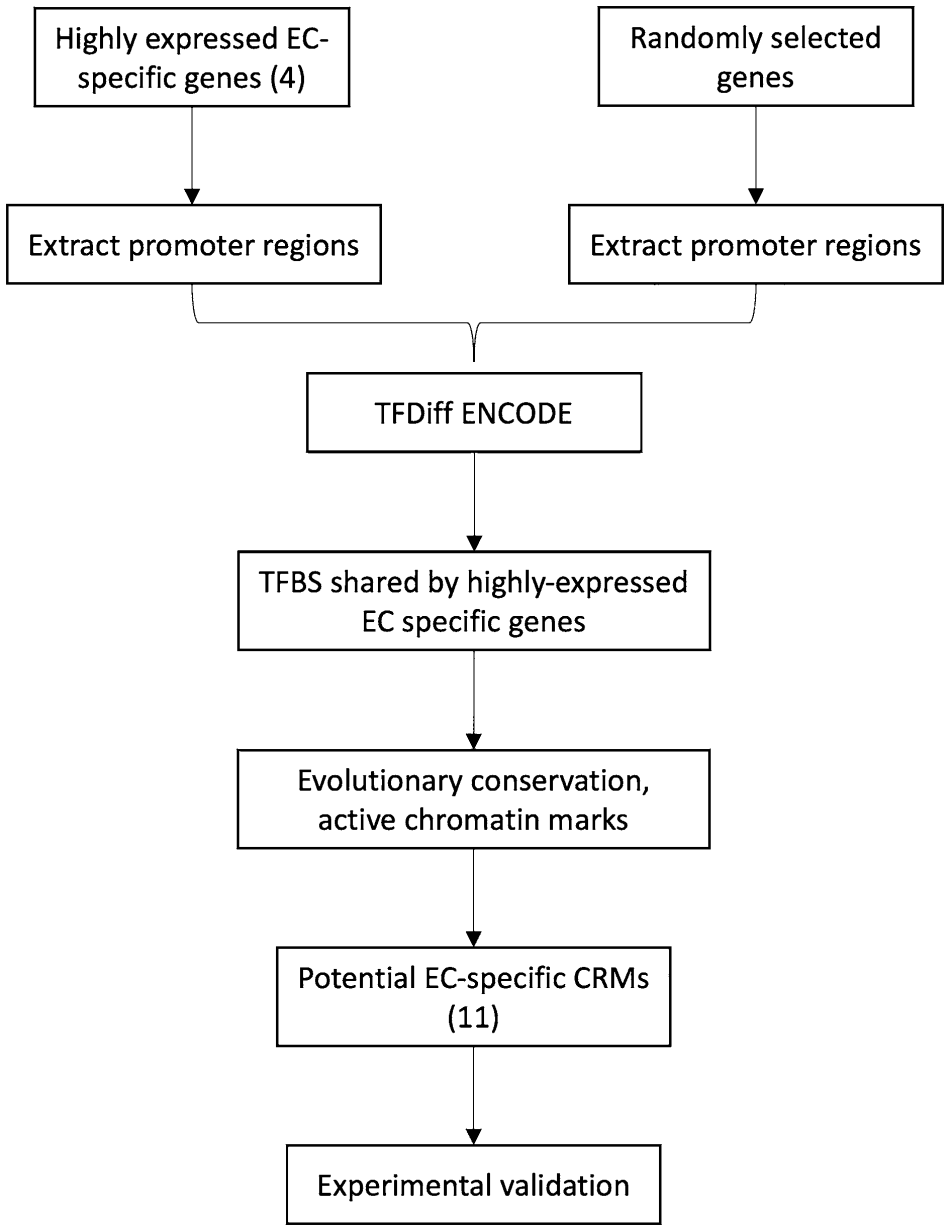
Bioinformatics-based approach used to identify and test 11 potential endothelial cell (EC)-specific cis-regulatory modules (CRM; see “Methods”). Essentially, promoter regions of 4 highly expressed EC genes were analyzed to identify sequences that bind transcription factors and are likely transcriptionally active. TFBS, transcription factor binding sites.

We next tested a bioinformatics-based approach, shown previously to be useful in generating high-expressing cell-type-specific expression cassettes for hepatocytes, cardiomyocytes, and skeletal myocytes^16–18^. This approach exploits short DNA sequences (cis-regulatory modules; CRM), identified as transcriptionally active based on their ability to bind transcription factors near promoters of genes that are highly expressed in specific cell types (Fig. 3). To identify EC-specific CRM, we first identified 8 transcription-factor binding sites (TFBS) that: (i) are preferentially associated with 4 highly expressed relatively EC-specific genes (*CDH5, EFEMP1*, *THBS1, VWF;* (https://www.ebi.ac.uk/arrayexpress/experiments/E-GEOD-26284/)^23^; and (ii) tend to co-occur in proximity to each other within evolutionarily conserved regions that are transcriptionally active in HUVEC (based on presence of open chromatin and histone marks of active transcription). These TFBS contain consensus binding-sites for the transcription factors AR, E2F7, ESR2, ETS1, GATA3, PRDM1, SNAPC1, and TAF7. By scanning within 2 kb of the transcription start sites of the 4 highly expressed relatively EC-specific genes, we identified 11 DNA segments that include clusters of these TFBS and might therefore represent EC-specific CRM. These 11 CRM (55–352 bp) are located in the promoters or introns of the 4 relatively EC-specific genes: *CDH5* (CRM 1–4), *EFEMP1* (CRM 5–7), *THBS1* (CRM 8–10), and *VWF* (CRM11) (Supplementary Fig. S2).

**Figure 4:**
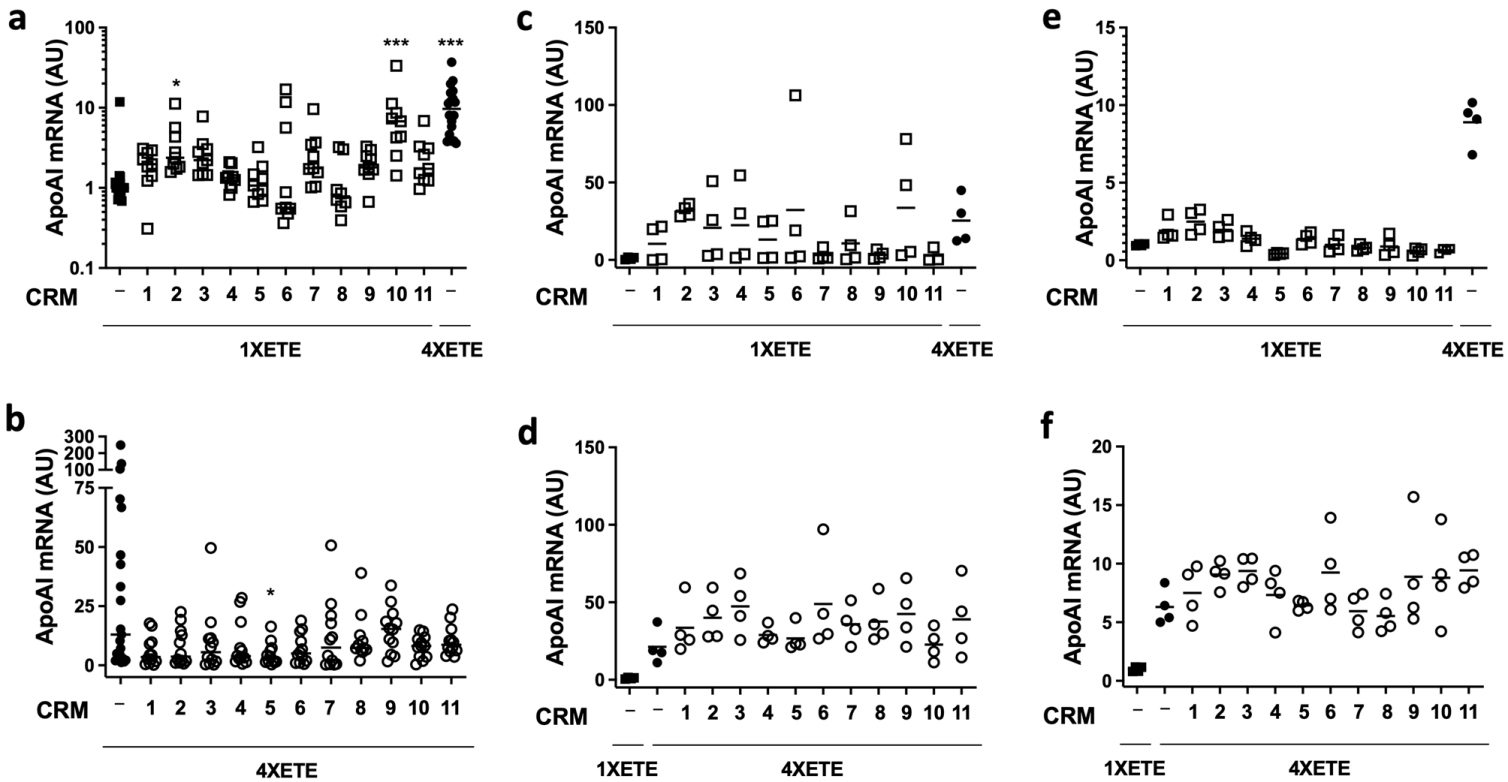
Testing of *APOAI* expression cassettes containing cis-regulatory modules (CRM) in endothelial cells. (**a**) Bovine aortic endothelial cells (BAEC) were transfected with pBshuttle-1XETE-gApoAI-oPRE either without an added CRM (−) or with one of the CRM identified via the process in Fig. 3. BAEC were also transfected with pBshuttle-4XETE-gApoAI-oPRE, without an added CRM. Median *APOAI* expression for 1XETE-gApoAI-oPRE is assigned a value of 1; all other points are measured relative to this value. (**b**) BAEC were transfected with pBshuttle-4XETE-gApoAI-oPRE without an added CRM (−) or with one of the CRM. (**c**) Human aortic endothelial cells (HAEC) were transfected with pBshuttle-1XETE-gApoAI-oPRE either without an added CRM (−) or with 1 of the CRM. HAEC were also transfected with pBshuttle-4XETE-gApoAI-oPRE, without an added CRM. (**d**) HAEC were transfected with pBshuttle-4XETE-gApoAI-oPRE without an added CRM (−) or with 1 of the CRM. HAEC were also transfected with pBshuttle-1XETE-gApoAI-oPRE, without an added CRM. (**e**) Human umbilical vein endothelial cells (HUVEC) were transfected with pBshuttle-1XETE-gApoAI-oPRE either without an added CRM (−) or with 1 of the CRM. HUVEC were also transfected with pBshuttle-4XETE-gApoAI-oPRE, without an added CRM. (**f**) HUVEC were transfected with pBshuttle-4XETE-gApoAI-oPRE without an added CRM (−) or with 1 of the CRM. HUVEC were also transfected with pBshuttle-1XETE-gApoAI-oPRE, without an added CRM. (**c**–**f**) Mean *APOAI* expression for 1XETE-gApoAI-oPRE is assigned a value of 1; all other points are measured relative to this value. (**a**–**f**) *APOAI* mRNA was measured by RT-qPCR, 24 h after transfection. Data are from three (**a**); four (**b**); or two (**c**–**f**) independent experiments; bars are group medians (**a**,**b**) or group means (**c**–**f**). (**a**) *P < 0.05; ***P < 0.001 vs. 1XETE-gApoAI-oPRE. (**b**) *P < 0.05 vs. 4XETE-gApoAI-oPRE.

We cloned each of the 11 CRM from human DNA and inserted them into both pBshuttle-1XETE-gApoAI-oPRE and pBshuttle-4XETE-gApoAI-oPRE, just upstream of the 1XETE and 4XETE promoter/enhancer sequences (Supplementary Fig. S1). Transfection of the 1XETE-based CRM-containing constructs into BAEC revealed that CRM2 and CRM10 significantly increased *APOAI* expression from 1XETE-gApoAI-oPRE (2-fold; *P* = 0.02 for CRM2 and 7-fold *P* < 0.001 for CRM10; Fig. 4a). In comparison, BAEC transfected in parallel with pBshuttle-4XETE-gApoAI-oPRE (without an added CRM) expressed 10-fold more *APOAI* mRNA than pBshuttle-1XETE-gApoAI-oPRE (*P* < 0.001). Transfection of the 4XETE-based CRM-containing constructs into BAEC revealed that none of the 11 CRM significantly increased *APOAI* expression from 4XETE-gApoAI-oPRE (Fig. 4b).

**Figure 5:**
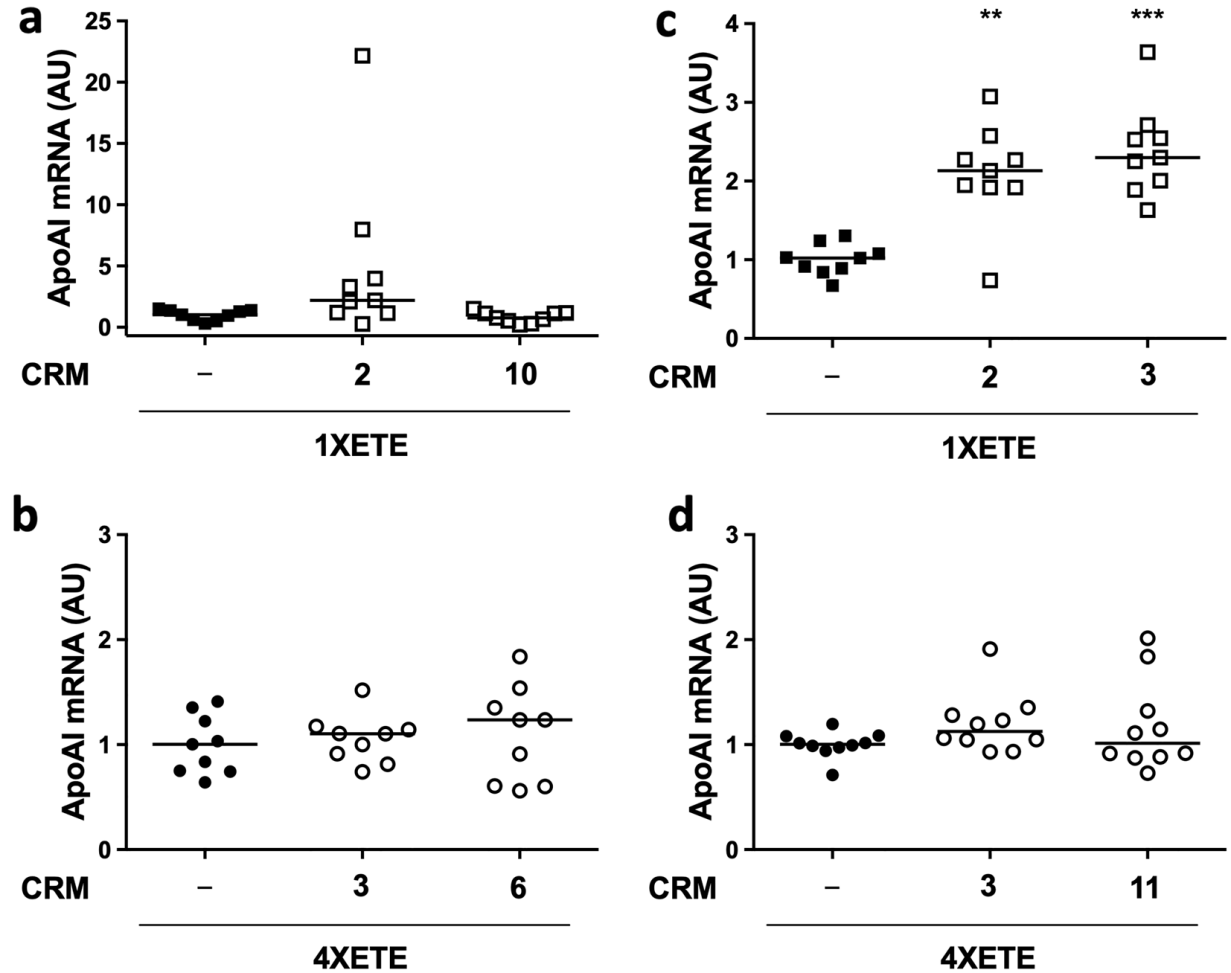
Testing of selected *APOAI* expression cassettes containing cis-regulatory modules (CRM) in human endothelial cells. (**a**) Human aortic endothelial cells (HAEC) were transfected with pBshuttle-1XETE-gApoAI-oPRE either without an added CRM (−), with CRM2, or with CRM10. (**b**) HAEC were transfected with pBshuttle-4XETE-gApoAI-oPRE without an added CRM (−), with CRM3, or with CRM6. (**c**) Human umbilical vein endothelial cells (HUVEC) were transfected with pBshuttle-1XETE-gApoAI-oPRE either without an added CRM (−), with CRM2, or with CRM3. (**d**) HUVEC were transfected with pBshuttle-4XETE-gApoAI-oPRE without an added CRM (−), with CRM3, or CRM11. (**a**,**c**) Median *APOAI* expression for 1XETE-gApoAI-oPRE is assigned a value of 1; all other points are measured relative to this value. (**b**,**d**) Median *APOAI* expression for 4XETE-gApoAI-oPRE is assigned a value of 1; all other points are measured relative to this value. (**a**–**d**) *APOAI* mRNA was measured by RT-qPCR, 24 h after transfection. Data are from three independent experiments; bars are group medians. (**c**) **P < 0.01; ***P < 0.001 vs. 1XETE-gApoAI-oPRE.

Because we cloned the CRM from human DNA, we considered that they might more-effectively bind transcription factors and increase *APOAI* expression in human rather than bovine cells. For this reason, we next tested the CRM in human aortic endothelial cells (HAEC). Because of technical challenges in expanding large numbers of HAEC, we began by screening all 11 CRM in 2 independent transfections. Based on these results (Fig. 4c,d), we selected CRM 2 and 10 for further testing with the 1XETE-gApoAI-oPRE backbone and CRM 3 and 6 for further testing with the 4XETE-gApoAI-oPRE backbone. In a larger series of transfections, however, none of these 4 CRM significantly altered expression from the corresponding backbones (Fig. 5a,b).

We next considered that the CRM were identified based on gene-expression data from cultured HUVEC (see “Methods”). Accordingly, we screened all 11 CRM in HUVEC, again with 2 separate transfections. Based on these results (Fig. 4e,f), we selected CRM 2 and 3 for further testing with the 1XETE-gApoAI-oPRE backbone and CRM 3 and 11 for further testing with the 4XETE-gApoAI-oPRE backbone. In a larger series of transfections, both CRM2 and CRM3 significantly increased expression from 1XETE-gApoAI-oPRE (2.1-fold; *P* < 0.01 and 2.3-fold; *P* < 0.001, respectively; Fig. 5c). Neither CRM3 nor CRM11 significantly altered expression from 4XETE-gApoAI-oPRE (Fig. 5d).

### Insertion of the *APOAI* gene at the transcription start site of highly expressed EC genes

We noted that CRM 2, 3, and 10 could increase *APOAI* expression from the 1XETE-gApoAI-oPRE backbone (Figs. 4a and 5c) but not from the 4XETE-gApoAI-oPRE backbone (in which the DNA sequences adjacent to the CRM and the distance from the CRM to the transcription start site differ). Moreover, all CRM were identified based on attributes (e.g., presence of open chromatin, transcription factor binding activity) that were measured in their native genomic contexts. Accordingly, we hypothesized that the CRM might function more optimally in their genomic contexts (i.e., when they are situated among native neighboring DNA sequence and are separated from nearby transcription start sites by the same number of nucleotides as in the human genome). To test this hypothesis, we cloned the CRM-containing genomic regions of *CDH5*, *EFEMP1*, *THBS1*, and *VWF* and inserted the rabbit *APOAI* gene into the translation start site of each of these 4 genes (Supplementary Fig. S2).

**Figure 6:**
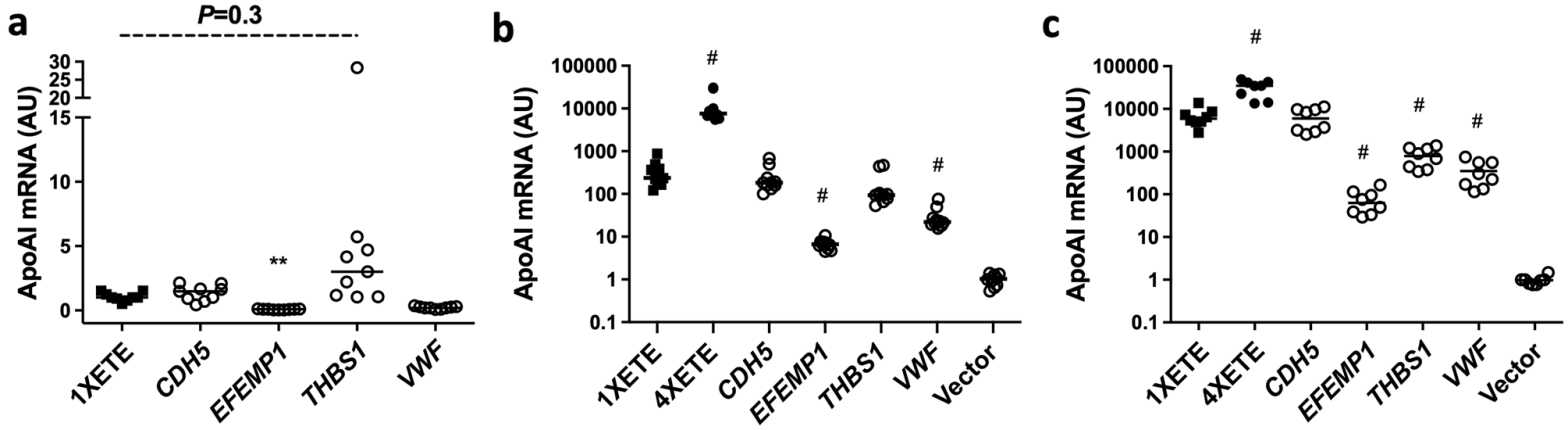
Expression of *APOAI* from genomic regions associated with highly expressed transcripts in cultured endothelial cells. (**a**) Bovine aortic endothelial cells were transfected with pBshuttle-1XETE-gApoAI-oPRE (1XETE) or with plasmids containing the rabbit *APOAI* gene inserted into genomic regions of *CDH5*, *EFEMP1*, *THBS1*, or *VWF* (see Supplemental Fig. S2). (**b**) Human aortic endothelial cells or (**c**) human umbilical vein endothelial cells were transfected with the same plasmids as in (**a**). Cells in (**b**,**c**) were also transfected with pBshuttle (vector), a plasmid that does not express *APOAI*. (**a**–**c**) *APOAI* mRNA was measured by RT-qPCR, 24 h after transfection. Data are from three independent experiments; bars are group medians. Median *APOAI* expression for 1XETE (**a**) or empty vector control (**b**,**c**) is assigned a value of 1; all other points are measured relative to this value. **P < 0.01; ^#^*P* < 0.005 vs. 1XETE.

When transfected into BAEC (Fig. 6a), the *CDH5*-based cassette expressed *APOAI* at a similar level to the 1XETE-gApoAI-oPRE construct (which is essentially a “knock-in” of the rabbit *APOAI* gene to a segment of the mouse *Edn1* locus, with the oPRE added; Supplementary Fig. S1). The *EFEMP1* and *VWF*-based cassettes expressed *APOAI* at a lower level than 1XETE-gApoAI-oPRE (92% less; *P* = 0.002 and 80% less; *P* = 0.09, respectively); whereas, the *THBS1*-based cassette expressed at a nominally higher level (3-fold; *P* = 0.3). To determine whether the *THBS1* result was falsely negative due to low statistical power associated with multiple comparisons, we performed new transfections that compared 1XETE-gApoAI-oPRE to THBS1-gApoAI-oPRE. We again found no significant difference in *APOAI* expression (*P* = 0.6; Supplemental Fig. S3).

Finally, for reasons described above, we tested the 4 knock-in cassettes in human EC: HAEC and HUVEC. We included an empty vector control (pBshuttle), to measure the absolute performance of all cassettes in these cell types. We included the 4XETE-gApoAI-oPRE cassette to compare the 4 new cassettes to our highest-expressing cassette^14,15^. When transfected into HAEC (Fig. 6b), all 4 of the new cassettes expressed *APOAI* at levels higher than the background signal in the vector-only control group (from ~ 6-fold for *EFEMP1* to ~ 180-fold for *CDH5*). However, none of the 4 cassettes expressed more *APOAI* mRNA than 1XETE-gApoAI-oPRE, with median expression ranging from ~ 3% (*EFEMP1*) to ~ 80% (*CDH5*) of 1XETE-gApoAI-oPRE levels. The *EFEMP1* and *VWF*-based cassettes expressed apoAI at a significantly lower level than 1XETE-gApoAI-oPRE (97% less; P < 0.0001; and 91% less; *P* < 0.01, respectively). In contrast, 4XETE-gApoAI-oPRE expressed apoAI at ~ 30-fold higher levels than 1XETE-gApoAI-oPRE.

When transfected into HUVEC (Fig. 6c), all 4 knock-in cassettes expressed *APOAI* far above the signal in the vector-only control group (from ~ 65-fold for *EFEMP1* to ~ 6000-fold for *CDH5*). Expression from the *CDH5*-based cassette was essentially identical to expression from 1XETE-gApoAI-oPRE; the other 3 cassettes expressed at far lower levels (1–13% of 1XETE-gApoAI-oPRE levels). Again, 4XETE-gApoAI-oPRE expressed higher levels of *APOAI* mRNA than 1XETE-gApoAI-oPRE (~ 6-fold; *P* < 0.005).

## Discussion

We tested several strategies aimed at increasing expression of a rabbit *APOAI* transgene in vascular endothelial cells. Our major findings are: (1) addition of an SSRE sequence to our previously highest-expressing expression cassette (4XETE-gAPOAI-oPRE) did not increase transgene expression in EC in vitro or in vivo. (2) Addition of 2 *Mef2c* enhancer elements significantly increased EC transgene expression from a moderately active expression cassette (1XETE-gAPOAI-oPRE) but not from 4XETE-gAPOAI-oPRE. (3) Similarly, we identified 3 CRM (of 11 potential EC-specific CRM) that increased EC transgene expression from 1XETE-gAPOAI-oPRE); however, none of the 11 CRM increased transgene expression from 4XETE-gAPOAI-oPRE. (4) Insertion of the rabbit *APOAI* gene into the transcription start sites of 4 highly expressed EC genes did not increase *APOAI* expression above 1XETE-gAPOAI-oPRE.

Houston et al. reported that an SSRE (GAGACC)—first identified in the platelet-derived growth factor promoter and conserved in several shear-stress-responsive genes—increased transgene expression from a heterologous promoter in response to shear stress both in vitro (3–8-fold) and in vivo (10-fold, in rabbit carotid arteries)^19^. This SSRE binds specifically to nuclear extracts of both static and shear-stressed EC, although the binding proteins were not identified^24^. Presumably, these binding proteins include transcription factors or co-regulators that are activated by shear, prompting us to add the SSRE to the 4XETE promoter/enhancer. Failure of the SSRE to increase *APOAI* transgene expression in shear stress-exposed EC in vivo might be explained by generation of binding site(s) for transcriptional repressors in the SSRE-4XETE construct, lack of synergy with factors bound to 4XETE, competition by the endogenous GAGACC sequence in 4XETE, or insufficient shear stress in our transduced carotid arteries.

Others have used EC-specific enhancers (e.g., from *NOS3* and *TEK*) to increase EC-specific transgene expression from heterologous promoters^25,26^. De Val et al. identified a highly conserved 44-bp enhancer sequence in the mouse *Mef2c* locus that was sufficient to drive EC-specific gene expression in vivo. Moreover, when placed upstream of a heterologous promoter and transfected in vitro, expression driven by this 44-bp sequence was upregulated 40-fold by co-transfection of the Foxc2 and Etv2 transcription factors^22^. This 44-bp sequence contains a FOX:ETS motif that is overrepresented in EC-expressed genes, increasing the likelihood that it would be active in multiple genomic contexts, including ours. Moreover, because EC enhancer activity can be increased by concatemerization^14,27^, we inserted 1–5 *Mef2c* enhancer copies upstream of our promoters. Addition of 2 copies of the 44-bp enhancer significantly increased expression from 1XETE; however, all the *Mef2c* enhancer constructs substantially decreased expression when placed upstream of 4XETE. Potential explanations include competition for transcription factor binding between the Mef2c enhancers and the 4 *Edn1*-derived enhancers in 4XETE or direct interactions between the ETE and Mef2c enhancers that interfere with transcription factor binding.

Cell type-specific CRM were initially identified by measuring highly expressed and lowly expressed genes in organ total-RNA extracts, then scanning promoter regions to identify—in the highly expressed genes—short DNA sequences containing TFBS that are over-represented and co-occur^16–18^. This approach identified CRM that—when fused to heterologous tissue-specific promoters—significantly increased in vivo transgene expression in liver, cardiomyocytes, and skeletal muscle. The activity of the CRM in these initial reports was impressive, increasing in vivo transgene expression from plasmids and AAV vectors by up to 200–400-fold and preserving tissue specificity^16–18^. However, this early work on CRM-containing vectors from the Chuah/VandenDriessche group and others^28–34^ also revealed limitations: (1) Some of the initially described CRM had no effect or decreased expression when placed upstream of the transthyretin promoter^16^. (2) CRM activity depends both on the identity of the adjacent promoter and on the transgene that is expressed, with up to 10-fold differences in CRM activity depending on the promoter^16–18,29^, and similar transgene-dependent variability^18^. (3) Several reports that used CRM did not compare CRM-containing vectors to vectors that differed only in lacking the CRM^29–31,33^. In one case, this comparison revealed that addition of a cardiomyocyte-specific CRM to the cardiac troponin T promoter either had no effect or decreased transgene expression in mouse hearts^32^. (4) When compared to enhancer-promoter combinations that lack identified CRMs, CRM-containing vectors have not always yielded the highest levels of expression^31^.

In the present study, we identified 3 EC-specific CRM (2, 3, and 10) that increased transgene expression from the 1XETE expression cassette. The most impressive results were obtained with CRM10 in BAEC (7-fold increase), yielding levels almost as high as the 4XETE cassette. However, CRM10 did not reliably increase transgene expression from the 1XETE cassette in the 2 other EC types and none of the CRM increased transgene expression from the 4XETE cassette. We considered several reasons for these results. First, in HAEC and HUVEC we performed 2 screening transfections of the 11 CRM, then focused on only 2 CRM. Additional transfections might have revealed high activity of the CRM that were screened out at the first step. However, this seems unlikely because when CRM boosted expression, they did so consistently (Figs. 4a and 5c). Second, we screened promoters of only 4 highly and lowly expressed genes, whereas other studies screened promoters of 29–59 highly expressed genes^16–18^. However, the optimal number of input genes for this approach is uncertain, and use of a larger number of genes in the ENCODE data set (Supplementary Table 1) to identify EC-specific CRM could risk obscuring signal with noise^18^. Third, we might have used suboptimal EC types to screen for CRM activity. We attempted to address this by including HUVEC, the same cell type in which the highly expressed genes were identified (see “Methods”). However, it is possible that differences in cell origin or culture conditions^35^ created a mismatch between the cellular transcriptional environment in which the CRM were discovered and the environment in which we tested the CRM. Fourth, the abundance of a transcription factor that binds to 4XETE may be limiting, blocking activity of added CRM.

In contrast to our in vitro-based approach, CRM active in hepatocytes, cardiomyocytes, and skeletal muscle were all identified using in vivo transcriptomes of whole liver, heart, and muscle^16–18^. A potential limitation of the in vivo approach is that transcripts in whole organ extracts could originate from any of several cell types. Because EC are a minority cell type in all organs, we decided at the outset of this project to use in vitro data (from purified EC) to identify highly expressed EC genes. Recent single-cell RNA sequencing data now provides access to the in vivo EC transcriptome^36,37^. Future efforts to identify EC-specific CRM should leverage these newly available data rather than relying, as we did, on data from cultured cells.

Insertion of a transgene into a locus with high cell-specific transcriptional activity is an effective means for achieving high levels of tissue-specific transgene expression^38^. Both the 1XETE and 4XETE cassettes are based on this premise because transgenes inserted into the *Edn1* locus (elements of which are included in 1XETE and 4XETE) are expressed at high levels in EC in vivo^39^. We attempted to generate cassettes with higher *APOAI* transgene expression than 4XETE by inserting the *APOAI* gene into vectors that contain core transcriptional elements of 4 other highly expressed EC genes. All vectors expressed *APOAI* mRNA far above background (up to 6000-fold for the CDH5-derived cassette in HUVEC; equivalent to 1XETE); however, none of the vectors expressed *APOAI* mRNA at a higher level than 1XETE and all expressed far less *APOAI* mRNA than 4XETE. We speculate that the high activity of 4XETE (which outperforms the CMV promoter in BAEC)^15^ might be explained by addition of engineered enhancer elements (3 *Edn1* enhancers) to the endogenous *Edn1* sequence.

Our study has limitations. First, some of our vectors were tested only in BAEC and only the SSRE-containing vector was tested in vivo. In our previous studies, expression cassette performance in BAEC has correlated well with performance in vivo in rabbit carotids^10,14,15^, but it remains possible that one or more of the cassettes described here could perform far better in vivo in rabbit EC than in vitro in BAEC. Second, we did not measure APOAI protein, and cannot rule out that a cassette might express the same or less mRNA as 4XETE-oPRE but more protein. However, the mRNA expressed from most of the constructs is identical (Supplemental Fig. S1) and all the mRNA have identical coding regions and 3′ untranslated regions (in which most posttranscriptional regulation takes place; Supplemental Figs. S1 and S2). Moreover, in past studies performed with interleukin 10-expressing vectors, secreted transgene protein levels correlated well with levels of transgene mRNA^14,15^. Third, our study is based on an untested assumption: that levels of APOAI are limiting in EC-mediated APOAI gene therapy. This can be tested only by experimentally increasing APOAI protein levels and measuring atherosclerosis.

In conclusion, we identified several sequences that are transcriptionally active in cultured EC. However, none of these sequences yielded an expression cassette with higher activity than 4XETE. Future strategies might include techniques such as Hi-C^40^ to identify distant sequences that transactivate highly expressed EC-specific genes, use of in vivo EC transcriptome data to identify other highly expressed EC genes and their CRM (with incorporation of these new CRM into expression cassettes), and insertion of transgenes into the loci of these newly identified EC genes.

## Methods

### Construction of expression cassettes and helper-dependent adenovirus (HDAd)

We constructed several expression cassettes and one HDAd (Supplemental Figs. S1 and S2). Details of cassette construction are available in the Supplementary Information.

### Identification of cis-regulatory modules (CRM)

To identify potential EC-specific CRM, we updated a computational approach that was used to identify liver-, cardiac-, and skeletal muscle-specific CRM^16–18^. As described above, we identified 4 target genes that are expressed highly and relatively specifically in EC (*VWF*, *EFEMP1*, *CDH5*, and *THBS1*). In the original approach to CRM identification, transcription factors were linked to target genes using transcription factor binding site (TFBS) predictions based on libraries of positional weight matrices; however, this approach is known to result in numerous false positives^41^.

Here we updated this approach to incorporate information contained in the ReMap 2015 ChIP-Seq database of 237 experimentally validated TFBS (http://tagc.univ-mrs.fr/remap/). This database, created by Griffon et al.^42^ uses systematic integration of public non-ENCODE and ENCODE experimental data^43^ to construct matrices that link TFBS to target genes, relying on the concept of regulatory potential (i.e., the likelihood that a gene is regulated by a transcription factor). Tang et al.^44^ modeled the influence of each TFBS on gene regulation as a function that decreases monotonically with increasing distance from the transcription start site of the gene. We used these tools to calculate—for each of the 237 transcription factors contained in the ReMap 2015 ChIP-Seq data sets—a regulatory potential on each of the 25,635 genes annotated in the human hg19 genome assembly. From the distribution of regulatory potential scores for every transcription factor, a p-value was calculated from the fraction of regulatory potential scores exceeding a given value. From these data, we constructed a general regulatory potential transcription factor–target gene matrix, in which genes are in rows and transcription factors are in columns. For every gene/transcription factor cell, the number 1 indicates if the gene is potentially regulated by the transcription factor at the specified p-value cutoff, the number 0 if it is not. For every specified p-value cutoff, a different general transcription factor–target gene matrix is constructed that can be used directly in the distance difference matrix approach, replacing the original positional weight matrices predictions-based transcription factor–target gene matrices.

We used the set of 4 genes that are expressed highly and relatively specifically in EC (*VWF*, *EFEMP1*, *CDH5*, and *THBS1*) and 1000 background sets of 8 randomly selected genes as input for the distance difference matrix method^45^. Because the background sets are generated randomly, we avoided sampling or selection bias by repeating the experiment 7 times. To identify transcription factors that are consistently found among the top regulatory factors in replicate experiments, we used a rank-product meta-analysis algorithm^46^. This yielded 8 top-regulatory transcription factors using a q-value cutoff of 0.05 (AR, E2F7, ESR2, ETS1, GATA3, PRDM1, SNAPC1, and TAF7). Clusters of TFBS associated with these factors were then used to identify putative CRM in the vicinity of the loci of the highly expressed, EC-specific genes.

In view of the limited number of target genes, putative CRM were identified visually with the UCSC Genome Browser and the GRCh37/hg19 assembly. For this purpose, a custom browser guiding track containing the binding regions for the 8 top regulators was generated and uploaded. We used additional tracks to help identify putative CRM including the layered HUVEC H3K4Me1 track (regulatory elements), the layered HUVEC H3K4Me3 track (promoters), the layered HUVEC H3K27Ac track (active regulatory elements), the DNase I Hypersensitivity tracks (open chromatin; regulatory regions and promoters), and the Vertebrate Multiz Alignment and Conservation (100 species) track (sequences maintained by natural selection). Using these tools, we identified putative CRM consisting of clusters of top-regulatory TFBS and coinciding with the strongest epigenetic modifications favoring transcription in HUVEC, indicators of open chromatin regions, and highly conserved sequence elements. This approach identified 11 potential EC-specific CRM (Supplementary Table S2). The source code is available at https://github.ugent.be/pdbleser/Tfdiff_REMAP.

### Testing expression cassettes in vitro

Bovine aortic EC (BAEC; Cell Applications Inc., San Diego, CA) were grown in Dulbecco’s Modified Eagle’s Medium (DMEM; Gibco, Grand Island, NY) supplemented with 10% fetal bovine serum (FBS) and 1% penicillin/streptomycin and were used at passages 5–8. Human aortic endothelial cells from a single donor, 37-year-old female, (HAEC; Lonza, Walkersville, MD) were grown in EBM-2 Basal Medium supplemented with EGM-2 SingleQuots Supplements (Lonza) and used at passages 4–7. Pooled Human Umbilical Vein Endothelial Cells (HUVEC; Lonza, Walkersville, MD), were grown in EGM-2 Basal Medium supplemented with EGM-2 SingleQuots Supplements (Lonza) and used at passages 1–6. We used BAEC for testing our constructs because of their adult large-artery origin and because they are easy to propagate and use at low passage. BAEC were used by others to evaluate the transcriptional activity of cis-acting human DNA sequences in vitro^24^. Use of BAEC is supported by our previous work on expression cassette development, in which we typically found excellent concordance between cassette performance in BAEC and cassette performance in vivo in rabbit carotid arteries^10,14,15^. In addition, in the present study we addressed the issue of cross-species reactivity of cis-acting sequences by also using human cells (HAEC and HUVEC) to test the transcriptional activity of selected constructs that contained cloned human DNA.

To test the mSSRE-containing expression cassette, BAEC were grown in 35-mm dishes to 80% confluence and transfected with plasmid DNA. These and all other transfections were done with jetPRIME transfection reagent (Polyplus, New York, NY), using the manufacturer’s protocol. Six hours after transfection, the cells were washed with DMEM and their medium was replaced. Fifty-one hours after transfection, the cells were harvested for RNA and DNA quantitation. To test the expression cassette containing an exogenous SSRE, BAEC were grown to 80% confluence in 6-well plates and transfected with plasmid DNA. Five hours after transfection, the cells were washed with DMEM and their medium was replaced. Twenty-four hours after transfection, the cells were harvested for RNA and DNA quantitation. In all cases, cells were harvested and lysed either by the addition of Buffer RLT Plus (Qiagen) + 2-mercaptoethanol (for RNA and/or DNA quantitation), the addition of Buffer RLT (Qiagen) + 2-mercaptoethanol (for RNA quantitation), or the addition of TRI Reagent (Zymo Research, Irvine, CA).

To test expression cassettes containing *Mef2c* oligomers, CRM, or genomic constructs, BAEC were grown to 80% confluence and transfected in 6-well plates. Five hours after transfection, the cells were washed twice with DMEM and 600 µL of DMEM was added to the wells. Twenty-four hours after transfection, the conditioned medium was removed, and the cells harvested for RNA and DNA quantitation. The CRM-containing expression cassettes and the genomic constructs were also transfected into HAEC and HUVEC, in 12-well plates. Four hours after transfection, the cells were washed with PBS, and 1 mL of fresh medium was added to the wells. Twenty-four hours after transfection, the cells were harvested for RNA and DNA quantitation.

We purified EC RNA and DNA with the AllPrep DNA/RNA Mini Kit (Qiagen, Germantown, MD). Alternatively, RNA and DNA were purified separately with the Direct-zol RNA Miniprep Kit (Zymo Research) and the DNeasy Blood and Tissue Kit (Qiagen). RNA was digested with DNase I (Thermo Fisher Scientific, Waltham, MA) to remove contaminating genomic DNA; RNA samples from HAEC and HUVEC were digested with both DNase I (New England Biolabs) and *Pvu*II (New England Biolabs) to remove contaminating genomic DNA. Use of *Pvu*II was required to ensure digestion of human *APOAI* genomic DNA. RNA was reverse-transcribed and amplified with the Luna Universal Probe One-Step RT-qPCR Kit (New England Biolabs). Alternatively, RNA was reverse-transcribed with the qScript Flex cDNA Kit (Quantabio) and the *APOAI* cDNA was measured by quantitative real-time PCR amplification using PerfeCTa FastMix II (Quantabio). *APOAI* mRNA levels were normalized to GAPDH mRNA levels, measured in cells from the same well. Normalized *APOAI* expression levels were further normalized for transfection efficiency, assessed by measuring plasmid DNA in extracts of cells in the same well (for BAEC) or of cells in a separate well transfected in parallel (for HAEC). Primers and probes for qPCR (Supplementary Table S3) were ordered from Integrated DNA Technologies. When Ct values for GAPDH or plasmid DNA suggested a failed PCR or failed transfection in a well, results from that well were omitted. These criteria, which resulted in exclusion of results from a small number of wells, were applied uniformly to all plasmids.

### In vivo gene transfer to rabbit carotid arteries

Rabbits were fed a standard laboratory diet (16% rabbit PLT, Albers Animal Feed, Bellevue, WA). After 1 week of acclimation to the animal facility, 16 male New Zealand White rabbits (3.0–3.5 kg, Western Oregon Rabbit Co., Philomath, OR) underwent surgical isolation of both common carotid arteries, followed by temporary occlusion, and local luminal infusion of either HDAd-SSRE-4XETE-gApoAI-oPRE (n = 8 rabbits) or the control vector HDAd-4XETE-gApoAI-oPRE (n = 8 rabbits; both vectors were infused at 2 × 10^11^ viral particles/mL diluted in DMEM) as previously described^21^. Rabbits were randomized to each vector group using a random number generator. The operator was aware of group allocation but the person analyzing the samples was blinded to group allocation. Exclusion criteria were limited to illness; no rabbits were excluded from surgery, and no samples were excluded from analysis. Carotid arteries were harvested either 3 or 28 days after gene transfer. Three spaced segments (proximal, mid, and distal) of each transduced artery were snap-frozen in liquid nitrogen for RNA analysis. Total RNA was extracted from these segments (RNeasy Mini Kit, Qiagen) and was quantified by Nanodrop spectrophotometer (Thermo Scientific, Wilmington, DE). *APOAI* mRNA (the single outcome variable) was measured by quantitative reverse transcriptase-mediated PCR^10^. All animal experiments were approved by the University of Washington Office of Animal Welfare. All methods were carried out in accordance with relevant guidelines and regulations, including the Institute for Laboratory Animal Research Guide for the Care and Use of Laboratory Animals. All methods are reported in accordance with ARRIVE guidelines.

### Statistics

For comparing two groups, we used Student’s *t* test if conditions of normality and equal variance were met. If not, we used a Mann–Whitney rank-sum test. For experiments that compared multiple groups (i.e., number of *Mef2c* enhancer copies, different CRM, or the 4 genomic knock-in constructs), we used the Kruskal–Wallis one-way ANOVA on ranks with Dunn’s method for post-hoc corrections for multiple comparisons. Statistical analyses were performed with SigmaStat.

### Ethical standards

All animal studies were approved by the Office of Animal Welfare of the University of Washington and were carried out in accordance with ARRIVE guidelines.

## Supplementary Information

Supplementary Information.

## Data Availability

The datasets generated during and/or analyzed during the current study are available from the corresponding author on reasonable request.
