# Supplementary material for: Novel expression cassettes for increasing apolipoprotein AI transgene expression in vascular endothelial cells
Source: Sci Rep. 2022 Dec 6;12:21079. doi: 10.1038/s41598-022-25333-9 (PMC9726828; doi:10.1038/s41598-022-25333-9)
Supplement: Supplementary file 1 — Supplementary Information. [file 41598_2022_25333_MOESM1_ESM.pdf]

Supplementary information, Sethuraman et al

Detailed Methods

Construction of expression cassettes and helper-dependent adenovirus (HDAd)

We previously reported construction of the plasmid pBshuttle-4XETE-gApoAI-oPRE and the corresponding HDAd vector HDAd-4XETE-gApoAI-oPRE.[1] Both constructs contain the rabbit *APOAI* gene driven by a modified murine *Edn1* promoter (termed 4XETE; includes the *Edn1* promoter and 3 added tandem repeats of the *Edn1* enhancer) along with the optimized posttranscriptional regulatory element (oPRE) of the woodchuck hepatitis virus[2] (Supplementary Fig. S1). We noted that the *Edn1* promoter contains a 5´-GAGACC-3´ sequence in the "noncoding" DNA strand. This sequence, reported to be a shear stress response element (SSRE), was originally identified in the platelet-derived growth factor B chain promoter[3] and was shown to function in vascular endothelial cells, when placed in the coding strand, upstream of synthetic promoters.[4] To enable testing of an SSRE in the coding strand, we first mutated the endogenous 5´-GAGACC-3´ sequence in the noncoding strand (QuikChange Lightning Site-Directed Mutagenesis Kit; Agilent Technologies, Santa Clara, CA) to 5´-ATGTCA-3´, yielding pBshuttle-4XETE-gApoAI-oPRE-mSSRE (Supplementary Figure S1). As described in Results, this plasmid was not useful, and all remaining plasmids were constructed with the noncoding 5´-GAGACC-3´ sequence intact.

We used site-directed mutagenesis to insert the 5´-GAGACC-3´ sequence into the coding strand of pBshuttle-4XETE-gApoAI-oPRE, just upstream of the 4XETE sequence. The new plasmid was named pBshuttle-SSRE-4XETE-gApoAI-oPRE (Supplementary Fig. S1). Homologous recombination was used, as described,[1] to transfer the SSRE-4XETE-gApoAI-oPRE sequence from pBshuttle-SSRE-4XETE-gApoAI-oPRE to the HDAd backbone plasmid pC4HSU (Microbix Biosystems, Toronto, Ontario, Canada).[5] The resulting plasmid was used, along with 293Cre4 cells and H14 helper virus,[6] to generate HDAd-SSRE-4XETE-gApoAI-

oPRE. Concentrated HDAd vector preparations, characterized as described,[1,7] were 1.4 to 2.8 × 10^{12} viral particles/mL with helper virus contamination <1% and E1A-containing genomes <1 in 10^{6} viral genomes.

To construct additional expression cassettes in which DNA sequences were tested for their ability to increase *APOAI* expression above levels obtained with the 4XETE-gApoAI-oPRE cassette, we first used site-directed mutagenesis to insert *Mlu*I and *Bgl*II restriction sites upstream of the 4XETE sequence in pBShuttle-4XETE-gApoAI-oPRE, generating pBShuttle-MB-4XETE-gApoAI-oPRE (Supplementary Fig. S1). We then constructed an oligomer containing a 44-bp murine *Mef2c* enhancer,[8] flanked by *Mlu*I and *Bgl*II restriction sites on one end and a *Bam*HI restriction site (Integrated DNA Technologies, Coralville, IA) on the other end. We ligated 1 copy of this oligomer into *Mlu*I-*Bgl*II-digested pBShuttle-MB-4XETE-gApoAI-oPRE, generating pBshuttle-1XMEF2C-4XETE-gApoAI-oPRE (Supplementary Fig. S1). We used the controlled and ordered oligonucleotide ligation procedure[9] to generate pBshuttle-2XMEF2C-4XETE-gApoAI-oPRE (Supplementary Fig. S1), as well as pBshuttle-3XMEF2C-4XETE-gApoAI-oPRE, pBshuttle-4XMEF2C-4XETE-gApoAI-oPRE, and pBshuttle-5XMEF2C-4XETE-gApoAI-oPRE (not shown).

To construct expression cassettes in which putative DNA enhancer sequences were tested for their ability to increase *APOAI* expression from a cassette containing only 1 copy of the *Edn1* enhancer (i.e., 1XETE-gApoAI-oPRE; Supplementary Fig. S1), we used site-directed mutagenesis to remove the 3 added copies of the *Edn1* enhancer (total 160 bp) from pBshuttle-MB-4XETE-gApoAI-oPRE, leaving the *Mlu*I and *Bgl*II sites intact. The product of this reaction, pBshuttle-MB-1XETE-gApoAI-oPRE (Supplementary Fig. S1) was used, along with the *Mef2c* oligomers mentioned above and the controlled and ordered oligonucleotide ligation procedure, to generate pBshuttle-1XMEF2C-1XETE-gApoAI-oPRE, pBshuttle-2XMEF2C-1XETE-gApoAI-oPRE, etc. (Supplementary Fig. S1; 3X 4X and 5XMEF2C constructs are not shown). We also used site-directed mutagenesis to delete the *Mlu*I and *Bgl*II restriction sites from pBshuttle-MB-

1XETE-gApoAI-oPRE, generating a control plasmid, pBshuttle-1XETE-gApoAI-oPRE (Supplementary Fig. S1).

We used a similar approach to construct expression cassettes in which single copies of endothelial cell (EC) cis-regulatory modules (CRM; identified as described below) were inserted upstream of the 4XETE and 1XETE sequences in pBshuttle-MB-4XETE-gApoAI-oPRE and pBshuttle-MB-1XETE-gApoAI-oPRE. Essentially, we used PCR and human DNA from 293 Cre cells[6] as a template to generate amplicons containing each of 11 CRM (all with introduced 5´ *Mlu*I sites and 3´ *Bgl*II sites), we digested the amplicons with *Mlu*I and *Bgl*II, and ligated the products into the *Mlu*I/*Bgl*II-digested plasmids. These expression cassettes were termed CRM(1–11)-1XETE-gApoAI-oPRE and CRM(1–11)-4XETE-gApoAI-oPRE (Supplementary Fig. S1).

We also constructed 4 expression cassettes in which *APOAI* expression was driven by large (2 035 – 8 880 bp) genomic segments of each of 4 genes that are highly and relatively specifically expressed in EC: *VWF*, *THBS1*, *EFEMP1*, and *CDH5.* To identify these 4 genes, we began by using publicly available search engines and PubMed, along with search terms that included "endothelium genes," "endothelium-specific genes," and "endothelial cell specific." These searches identified 30 candidate genes (Supplemental Table 1). We consulted the relevant publications and confirmed that each publication reported expression of these genes in EC. We then consulted the Gene Expression Atlas (http://www.ebi.ac.uk/gxa/home), which includes quantitative transcription data generated from the ENCODE project.[10] For each of the 30 genes, we interrogated ENCODE-derived data in the Gene Expression Atlas using: endothelial cell-derived cell line, the gene name, ENCODE – long polyA RNA, and whole cell. The output of this interrogation yielded a table that reports relative expression levels of the 30 genes in 18 cell lines, including cultured human umbilical vein EC (HUVEC; Supplementary Table S1). *EFEMP1* and *THBS1* were by far the most highly expressed genes in HUVEC and had relatively EC-specific expression. *VWF* and *CDH5* were the next most highly expressed

genes in HUVEC and were expressed only in HUVEC.

To determine which genomic sequences to incorporate in the 4 expression cassettes, we consulted papers that identified cis-acting positive regulators of transcription located near the promoters of the 4 genes.[11-21] We were also careful to include the genomic regions in which the CRM were located (see below). Into these genomic sequences, we ligated a cassette that contains elements of the rabbit *APOAI* gene (beginning with the start codon, and including all 3 coding exons, both introns, and 51 bp of 3´ untranslated region), as well as the oPRE and SV40 polyadenylation signal. This *APOAI* cassette was inserted at the translational start sites of each of these 4 genomic sequences (Supplementary Fig. S2). The *VWF* genomic sequence extends from 843 bp upstream of the transcription start site to the translation start site, including the promoter, first exon, and first intron (total 2 322 bp). We used 2 segments of the *EFEMP1* gene, with one segment placed upstream and one segment downstream of the *APOAI* gene. The upstream sequence extends from 243 base pairs upstream of the *EFEMP1* transcription start site to immediately upstream of the *EFEMP1* start codon, including the promoter, first exon, first intron, and part of the second exon (total 1 942 bp). The 3´ segment begins immediately downstream of the *EFEMP1* start codon, extends for 5 676 base pairs downstream of the *EFEMP1* translation start site (including the 2nd, 3rd, and 4th exons, the 2nd and 3rd introns, and part of the 4th intron). We used 2 separate segments of the *EFEMP1* gene because CRM were identified both 5´ and 3´ of the *EFEMP1* translation start site, and because leaving exon 2 (containing the *EFEMP1* transcription start site) intact would potentially result in transcription and translation of a chimeric mRNA that included both *EFEMP1* and *APOAI* sequences. The *THBS1* genomic sequence extends from 1 270 bp upstream of the transcription start site to the translation start site, including the promoter, first exon, first intron, and part of the second exon (total 2 035 bp). The *CDH5* genomic sequence includes 2 segments. The first segment extends from 6 721 bp upstream of the transcription start site through part of the first intron (total 8 821 bp). The second segment includes a sequence from

the 3´ end of the first intron, including the splice acceptor site, and part of the second exon (total 59 bp). A plasmid containing the second segment for *CDH5* and part of the 3´ end of the first segment was constructed by Integrated DNA Technologies.

We cloned these genomic regions by PCR amplification (Q5 High-Fidelity DNA Polymerase, New England Biolabs, Ipswich, MA) of human genomic DNA (Promega, Madison, WI) or of the human *CDH5* gene-containing plasmid constructed by Integrated DNA Technologies. Primers were designed based on the human GRCh38/hg38 genome assembly. We constructed plasmids containing the *VWF*, *EFEMP1,* and *CDH5* genomic sequences by ligation of PCR-amplified human genomic DNA to the rabbit *APOAI* gene and pBshuttle, using Gibson Assembly kits (New England Biolabs; Quantabio, Beverly, MA; GenScript, Piscataway, NJ). pBshuttle-THBS1-gApoAI-oPRE was constructed by GenScript. Plasmid identities were confirmed by restriction digestion (all plasmids) and by sequencing either across all the Gibson Assembly junctions or across the entire insert (pBshuttle-THBS1-gApoAI-oPRE).

References

1 Dronadula, N. *et al.* Construction of a novel expression cassette for increasing transgene expression in vivo in endothelial cells of large blood vessels. *Gene Ther.* **18**, 501–508 (2011).

2 Zufferey, R., Donello, J. E., Trono, D. & Hope, T. J. Woodchuck hepatitis virus posttranscriptional regulatory element enhances expression of transgenes delivered by retroviral vectors. *J. Virol.* **73**, 2886–2892 (1999).

3 Resnick, N. *et al.* Platelet-derived growth factor B chain promoter contains a cis-acting fluid shear-stress-responsive element. *Proc. Natl. Acad. Sci. U. S. A.* **90**, 4591–4595 (1993).

4 Houston, P., White, B. P., Campbell, C. J. & Braddock, M. Delivery and expression of fluid shear stress-inducible promoters to the vessel wall: applications for cardiovascular

gene therapy. *Hum. Gene Ther.* **10**, 3031–3044, doi:10.1089/10430349950016429 (1999).

5 Parks, R. J. *et al.* A helper-dependent adenovirus vector system: Removal of helper virus by Cre-mediated excision of the viral packaging signal. *Proc. Natl. Acad. Sci. U. S. A.* **93**, 13565–13570 (1996).

6 Chen, L., Anton, M. & Graham, F. L. Production and characterization of human 293 cell lines expressing the site-specific recombinase Cre. *Somat. Cell Mol. Genet.* **22**, 477–488 (1996).

7 Mittereder, N., March, K. L. & Trapnell, B. C. Evaluation of the concentration and bioactivity of adenovirus vectors for gene therapy. *J. Virol.* **70**, 7498–7509 (1996).

8 De Val, S. *et al.* Combinatorial regulation of endothelial gene expression by ets and forkhead transcription factors. *Cell* **135**, 1053–1064, doi:S0092-8674(08)01387-1 [pii]10.1016/j.cell.2008.10.049 (2008).

9 Blachinsky, E., Marbach, I., Cohen, R., Grably, M. R. & Engelberg, D. Procedure for controlling number of repeats, orientation, and order during cloning of oligonucleotides. *Biotechniques* **36**, 933–936, doi:10.2144/04366BM02 (2004).

10 Djebali, S. *et al.* Landscape of transcription in human cells. *Nature* **489**, 101–108, doi:10.1038/nature11233 (2012).

11 Blancafort, P., Magnenat, L. & Barbas, C. F., 3rd. Scanning the human genome with combinatorial transcription factor libraries. *Nat. Biotechnol.* **21**, 269–274, doi:10.1038/nbt794 (2003).

12 Blackburn, J., Tarttelin, E. E., Gregory-Evans, C. Y., Moosajee, M. & Gregory-Evans, K. Transcriptional regulation and expression of the dominant drusen gene FBLN3 (EFEMP1) in mammalian retina. *Invest. Ophthalmol. Vis. Sci.* **44**, 4613–4621, doi:10.1167/iovs.03-0112 (2003).

13 Chandler, R. L. *et al.* ARID1a-DNA interactions are required for promoter occupancy by SWI/SNF. *Mol. Cell. Biol.* **33**, 265–280, doi:10.1128/MCB.01008-12 (2013).

14 Yang, Q. W. *et al.* Methylation-associated silencing of the thrombospondin-1 gene in human neuroblastoma. *Cancer Res.* **63**, 6299–6310 (2003).

15 Sahu, S., Ganguly, R. & Raman, P. Leptin augments recruitment of IRF-1 and CREB to thrombospondin-1 gene promoter in vascular smooth muscle cells in vitro. *Am J Physiol Cell Physiol* **311**, C212–224, doi:10.1152/ajpcell.00068.2016 (2016).

16 Nicklin, S. A. *et al.* Analysis of cell-specific promoters for viral gene therapy targeted at the vascular endothelium. *Hypertension* **38**, 65–70 (2001).

17 Othman, M. *et al.* Functional characterization of a 13-bp deletion (c.-1522_-1510del13) in the promoter of the von Willebrand factor gene in type 1 von Willebrand disease. *Blood* **116**, 3645–3652, doi:10.1182/blood-2009-12-261131 (2010).

18 Liu, J. *et al.* Vascular bed-specific regulation of the von Willebrand factor promoter in the heart and skeletal muscle. *Blood* **117**, 342–351, doi:10.1182/blood-2010-06-287987 (2011).

19 Guan, J., Guillot, P. V. & Aird, W. C. Characterization of the mouse von Willebrand factor promoter. *Blood* **94**, 3405–3412 (1999).

20 Bonthron, D. & Orkin, S. H. The human von Willebrand factor gene. Structure of the 5' region. *Eur. J. Biochem.* **171**, 51–57, doi:10.1111/j.1432-1033.1988.tb13757.x (1988).

21 Jahroudi, N. & Lynch, D. C. Endothelial-cell-specific regulation of von Willebrand factor gene expression. *Mol. Cell. Biol.* **14**, 999–1008, doi:10.1128/mcb.14.2.999 (1994).

Supplementary Figure S1

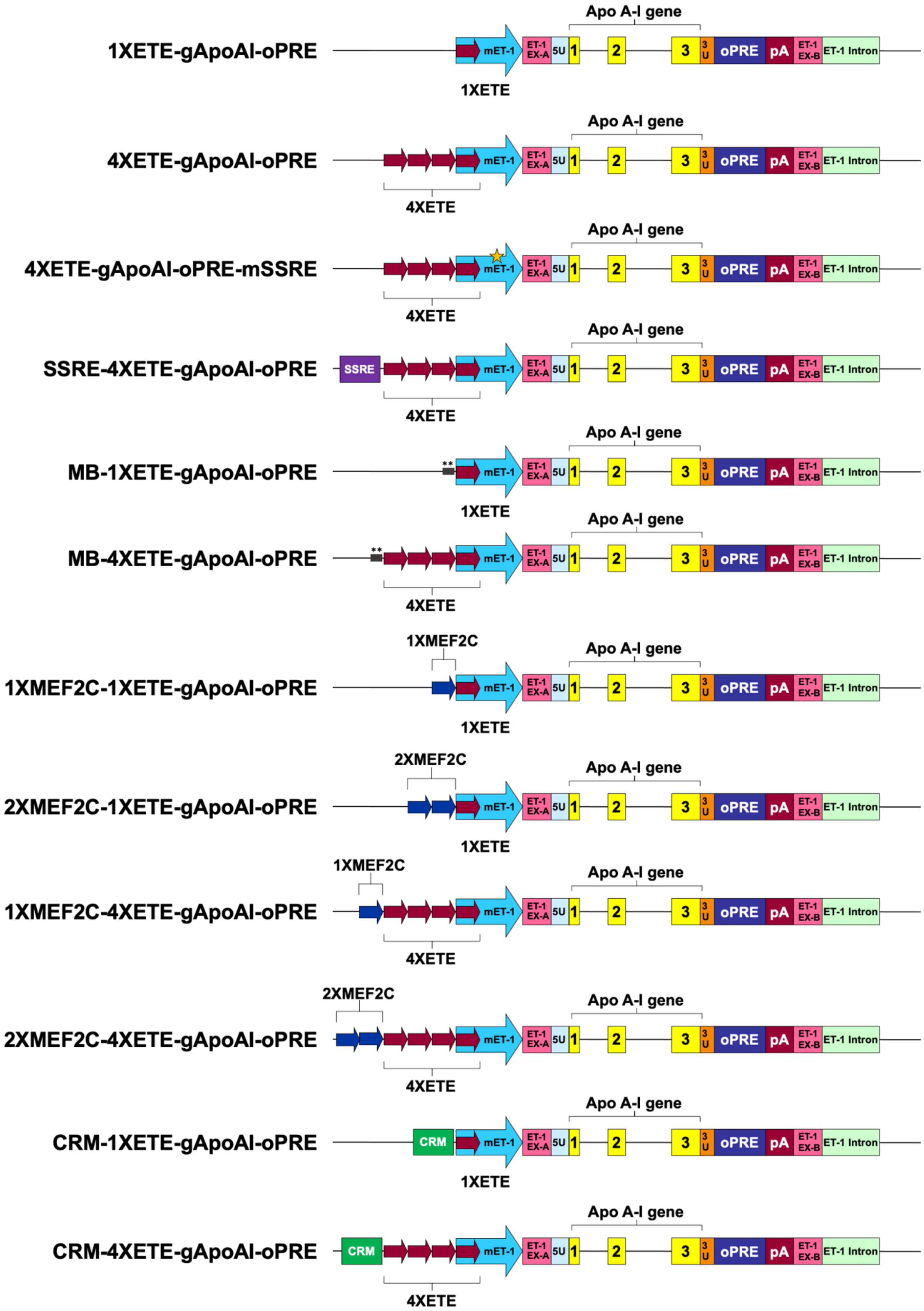

Supplementary Figure S1. Expression cassettes. All cassettes were tested in pBshuttle-based plasmids in vitro. Two of the cassettes, 4XETE-gApoAI-oPRE and SSRE-4XETE-gApoAI-oPRE were incorporated into helper-dependent adenoviral vectors and were also tested in vivo. Yellow blocks (1, 2, and 3), rabbit *APOAI* coding exons; large light-blue arrow (mET-1), murine endothelin-1 (*Edn1*) enhancer/promoter; red arrows, *Edn1* enhancer (1XETE), both in native position (within light-blue arrows) and added as 3 direct repeats (4XETE); yellow star, endogenous shear stress response element (SSRE) sequence; purple box, exogenous SSRE; ** introduced *Mlu*I and *Bgl*II restriction sites; small dark-blue arrow, mouse *Mef2c* enhancer element; green box, cis-regulatory module (CRM); 5U, 5´-untranslated region of rabbit *APOAI* gene, including exon 1 and intron 1; 3U, 3´-untranslated region of rabbit *APOAI* gene; ET-1 EX-A and ET-1 EX-B, 5´ and 3´ segments of mouse *Edn1* exon 1 (untranslated); ET-1 Intron, 5´ segment of mouse *Edn1* intron 1; oPRE, optimized woodchuck hepatitis virus post-transcriptional regulatory element; pA, SV40 poly A signal. Drawing is not to scale. Six other cassettes (3X, 4X, and 5XMEF2C-1XETE-gApoAI-oPRE and 3X, 4X, and 5XMEF2C-4XETE-gApoAI-oPRE) are not shown.

Supplementary Figure S2

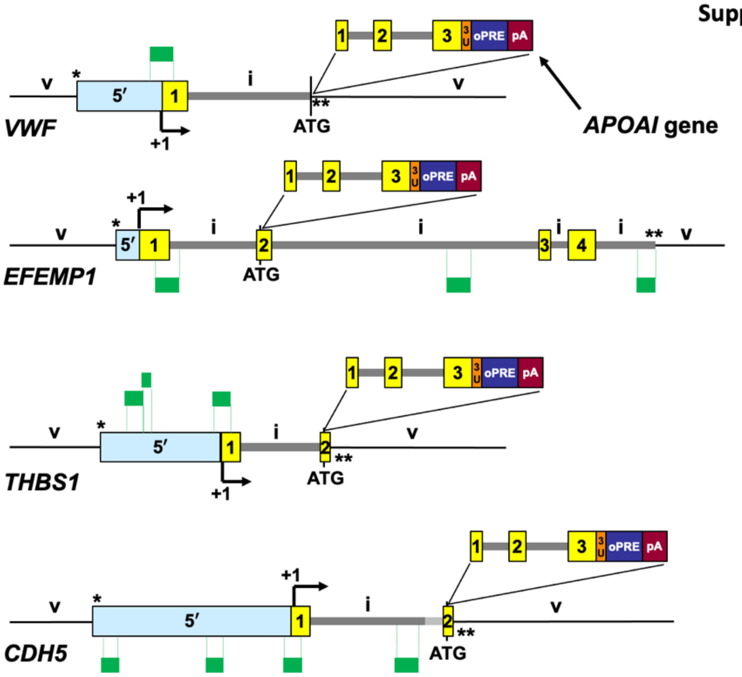

Supplementary Figure S2. Genomic knock-in expression cassettes. An engineered rabbit *APOAI* gene was inserted at the translation start sites (ATG) of genomic sequences cloned from the human *VWF*, *EFEMP1*, *THBS1*, and *CDH5* loci. The engineered *APOAI* gene includes the 3 *APOAI* coding exons (yellow boxes), beginning with the ATG in native exon 2, 2 *APOAI* introns (grey bars), a segment of *APOAI* 3´ untranslated region (3U), the optimized woodchuck hepatitis virus post-transcriptional regulatory element (oPRE), and an SV40 poly A signal (pA). Cloned segments of human genomic DNA in the cassettes include: sequences 5´ to the transcription start sites (blue boxes), the transcription start sites (+1), exons of *VWF*, *EFEMP1*, *THBS1*, and *CDH5* (yellow boxes numbered 1–4), introns (i), and the translation start sites (ATG). Green bars indicate positions of cis-regulatory modules identified in silico (see Materials and Methods). All cassettes were constructed in the pBshuttle plasmid vector (v). Junctions of the expression cassettes and pBshuttle are indicated (* and **). Drawing is not to scale.

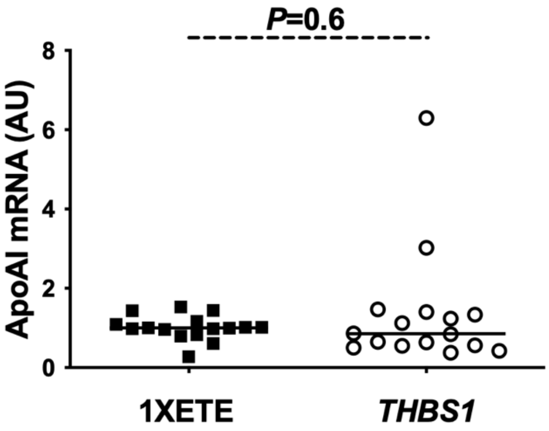

Supplementary Figure S3. *APOAI* expression from pBshuttle-1XETE-gApoAI-oPRE (1XETE) and pBshuttle-THBS1-gApoAI-oPRE (*THBS1*). Bovine aortic endothelial cells were transfected and *APOAI* mRNA was measured by RT-qPCR 24 hours after transfection. Data points are from four independent experiments; bars are group medians. Median *APOAI* expression for 1XETE-gApoAI-oPRE is assigned a value of 1; all other points are measured relative to this value.

Supplementary Table S1. Relative transcript levels for 29 endothelial cell (EC)-associated genes, from ENCODE.

	Cell Lines										
**Gene Name**	**Gene Symbol**	A549	AG445	BJ	CD20-positive B cell line	GM12878	H1-hESC	HMEC cell line	HSMM cell line	HUVEC cell line	PMID
EGF Containing Fibulin Extracellular Matrix Protein 1	EFEMP1	14	100	320			6	67	225	2379	12963823
Thrombospondin 1	THBS1	15	277	1522			5	398	912	1903	12963823
Von Willebrand Factor	VWF									582	23990205
Cadherin 5	CDH5									371	24003143
Endothelin 1	EDN1	52	1	2	1	5		2	10	329	12963823
EGF Like Domain Multiple 7	EGFL7	3	0.7	5			18	2	0.9	320	15972971
Intercellular Adhesion Molecule 2	ICAM2	0	0	0.1	16	6	0.1	0	0.1	258	12963823
Platelet And Endothelial Cell Adhesion Molecule 1	PECAM1				8	7				216	23990205
Protein C Receptor	PROCR	43	33	14			24	40	3	193	9396465
Endothelial Cell-Specific Molecule 2	ECSCR						2			188	18394197
Sphingosine-1-Phosphate Receptor 1	S1PR1		15	5	25	2	3	6	19	176	12963823
Tyrosine Kinase With Immunoglobulin Like And EGF Like Domains 1	TIE1					0.8	0.7			166	12963823
Activin A Receptor Like Type 1	ACVRL1		3	3					5	130	21467543
Multimerin 1	MMRN1					3				81	18394197
Roundabout Guidance Receptor 4	ROBO4							1	1	78	24003143
Endothelial Cell Specific Molecule 1	ESM1			0.6						72	12963823
Hedgehog Interacting Protein	HHIP									55	18394197
Kinase Insert Domain Receptor	KDR						38			54	11076864
Adhesion G Protein-Coupled Receptor L4	ELTD1		8	1					3	45	12963823
Tissue Factor Pathway Inhibitor	TFPI	18	11	3		3	0.6		31	34	16165014
Histone Deacetylase 7	HDAC7	2	3	8	20	0.6	9	6	7	27	24003143
Nitric Oxide Synthase 3	NOS3						2			18	23990205
TEK Receptor Tyrosine Kinase	TEK		19				7		3	17	24003143
Endomucin	EMCN									16	18394197
Intercellular Adhesion Molecule 1	ICAM1	0.6	14	18	17	26	3	1	0.9	14	22723222
Thrombomodulin	THBD	15						2	8	13	16165014
Endothelial Cell-Specific Molecule 2	ECSCR*									1	18394197
E-Selectin	SELE									0.9	22723222
Vascular Cell Adhesion Molecule 1	VCAM1		11			5			10	0.7	22723222
P-Selectin	SELP				2					0.7	17272818
*paralog											

Supplementary Table S1 (continued).

	Cell Lines										
**Gene Name**	**Gene Symbol**	HeLa-S3	HepG2	IMR-90	K562	MCF-7	NHEK cell line	NHLF cell line	SK-N-SH	SK-N-SH_RA	PMID
EGF Containing Fibulin Extracellular Matrix Protein 1	EFEMP1	28		68	1	8	62	161	395	26	12963823
Thrombospondin 1	THBS1	0.6	16	458		61	322	440	47	24	12963823
Von Willebrand Factor	VWF										23990205
Cadherin 5	CDH5										24003143
Endothelin 1	EDN1	1	0.6	6		4	13	4	1	1	12963823
EGF Like Domain Multiple 7	EGFL7	0.7	2	2	50	1	1	0.6	12	15	15972971
Intercellular Adhesion Molecule 2	ICAM2	0	2	0	5	0	0	0.1	0	0.2	12963823
Platelet And Endothelial Cell Adhesion Molecule 1	PECAM1		1		3						23990205
Protein C Receptor	PROCR	10	1	28	3	1	34	13	18	11	9396465
Endothelial Cell-Specific Molecule 2	ECSCR			1					3	2	18394197
Sphingosine-1-Phosphate Receptor 1	S1PR1			8			2	3	4	27	12963823
Tyrosine Kinase With Immunoglobulin Like And EGF Like Domains 1	TIE1			2						1	12963823
Activin A Receptor Like Type 1	ACVRL1			2				4			21467543
Multimerin 1	MMRN1										18394197
Roundabout Guidance Receptor 4	ROBO4										24003143
Endothelial Cell Specific Molecule 1	ESM1			1					10		12963823
Hedgehog Interacting Protein	HHIP			1					0.8		18394197
Kinase Insert Domain Receptor	KDR										11076864
Adhesion G Protein-Coupled Receptor L4	ELTD1			3				5			12963823
Tissue Factor Pathway Inhibitor	TFPI	13	52	5	58	0.7		29		0.8	16165014
Histone Deacetylase 7	HDAC7	2	2	12	5	3	3	5	10	6	24003143
Nitric Oxide Synthase 3	NOS3		0.7		2						23990205
TEK Receptor Tyrosine Kinase	TEK			4							24003143
Endomucin	EMCN					1					18394197
Intercellular Adhesion Molecule 1	ICAM1	3	42	8	6	0.8		4	8	42	22723222
Thrombomodulin	THBD					4	3	2			16165014
Endothelial Cell-Specific Molecule 2	ECSCR*										18394197
E-Selectin	SELE										22723222
Vascular Cell Adhesion Molecule 1	VCAM1			3				2	2	9	22723222
P-Selectin	SELP										17272818
*paralog											

Relative transcript levels are listed for the 29 genes in the 18 ENCODE cell lines, including human umbilical vein EC (HUVEC). Expression Atlas ID: E-GEOD-26284. PMIDs identify reports of expression of each gene in EC.

Supplementary Table S2. Sequences of cis-regulatory modules (CRM).

Name	Gene	Length	Sequence
CRM1	*CDH5*	161	AGGCAGCCGCCCACCGCAGGGCCTGCCTATCTGCAGCCAGCCCAGCCCTCACAA<br>AGGAACAATAACAGGAAACCATCCCAGGGGGAAGTGGGCCAGGGCCAGCTGGAA<br>AACCTGAAGGGGAGGCAGCCAGGCCTCCCTCGCCAGCGGGGTGTGGCTCCCCT
CRM2	*CDH5*	167	TTGCTTCCTCCTCTGCTACTAATCTGGTCTCACAGACCATCCCATTTCCTGCTAGC<br>CCACCAGCCGCCTTCCTTGCTCCCAATGACACTTCCTGGCCTTGTGCCCTCCTGTT<br>ACCTCCTTTGCCTCCAGAGAGGTTGGAGCAGAGGCTGGGCAGTGCCAGAAATCAG
CRM3	*CDH5*	268	CTGCCACACTGCCCAGCTGAGGGCTGGTGCCAGAGCCGTGTCTGCTTGCCCCAT<br>CAAGAGGTGGGAGGGATTGATCCACCTTCCTGCCCCACAGATGGTGCAGCCTCCA<br>ACCTATTGTTTTCCAGGACGCTTCGGTGGAGAGCACAAGGAATGTAGGGTCTAGA<br>AACAGGAAGCCCTGGCTTCCGCTGGACAAGGTTTCCTCCAGACTCAGGCCTGCCC<br>TCCAGACAACAAGGCAGGGCCCTTGGTCCCACCCTGCCCTGCCTGGCTC
CRM4	*CDH5*	332	GCCTGGAAGGGGTTAAGCTGCCCGCCTGCAGCCTCAGCCTGAGCTATTGTGTTGC<br>CAAACAAGGGCCGGACATGAGGGCAGGAAGCCAGCAGGGGCCACACATTTTCTG<br>CAAAGTTGGATGATTCACTGCTGACTTGGGGACACCCAGGGGACAGAGGGGACA<br>CCATCCCAGGAAGAATCTTAGGCTCATTTTGCCCACATGGACCCATGACTGTTCCC<br>TGTATCCTCTCTCTGCACCCCCTCAGTCACACTGAAGCAACTATGAGAATTCCCAT<br>TTGACAGATGGGACCATCGAGGCTGAGGGAAGCTGTGCAGCCAGTCCAAGGTCA<br>CAC
CRM5	*EFEMP1*	352	ACCCTGCACAGTCCAGGGCAGTTCTCGGGTACTCAACACCCCTCAGCTCACCCCA<br>CCTCACTCTCCCGCGCGCGGCCCAGTGAGTACTGGGCTCGCTCGGGGCGACCCC<br>CCGTTGGGGGCTCCTACCTGTGCGGCCGCGCTGCGCTCCGGGCCCGGGCAGCG<br>AGGGGAGTGCGCAGGGGAGGGCAGCCCCGTGGGTCTGATCTGGCGAAGTCCGG<br>CAGCGGCCGCGGCGGCAGGAGGAGGAGGAGAAAGGGAGGGAAAGGGGGAGGG<br>CGAAGGGGGGCGGAGGAGAGAGCTGAGGGAGGCGCGAGCGCTGAGCCCAGCGT<br>TGCGAGCCCGGCGGTAGAGCGCGCAGCGCAGC
CRM6	*EFEMP1*	345	TTGAGAAATGTTGCTACAGGCGATAAGAGGCCTCTGAAGAATTTTTAAGCTCAGGA<br>AACAATCTGATCAGACGCTCATTTGAGAAAGTCACTGTAAGGAAGAAGCCCAAGGA<br>AGGATTGAGCAAAAGTGGAGTAAACAAACAAATAAACTTAAATTCTTTTAATATAAC<br>TGAGAGCTGCTTATGTACACAATGGATGCTTGACAAGGAAATAGCTGAATGGAAGC<br>CCAGAGATCCATTTAGATATTTTACTGATGTCTGAAACTTACTGGAGCGAGAACTAA<br>ATTCACTTTGTCTCTCAGACTAATTTTTTTCTTGTTTCCCAAACAGTGTTAAAGGTAC<br>CACTA
CRM7	*EFEMP1*	272	AGTCTCCACACCAGGCTGCTCTGAAATGGTTTGCCAAGAAGAAAGGAAACAATCT<br>CATAGCACAGTTCAGTTAGCTAATATCCTACATGTCTTCAAAGCCCCAGATTCTTAT<br>CACTTTGCTAATACGAGCCTACAGCTGTTTCTAGAATACACAGTGATGTGTATGCT<br>CTGTGAAAATAAACAGAAAACCCTAGGCAGATGATGCCATGGTGCAAGTGATATAA<br>GTGAAACGTCGTCAAATGAGAGCTTAAAAGAGATATAAACGTGAGTG
CRM8	*THBS1*	198	CCCGCCCCCTTCACTTTCTAGCTGGAAAGTTGCGCGCCAGGCAGCGGGGGGCGG<br>AGAGAGGAGCCCAGACTGGCCCCCACCTCCCGCTTCCTGCCCGGCCGCCGCCCA<br>TTGGCCGGAGGAATCCCCAGGAATGCGAGCGCCCCTTTAAAAGCGCGCGGCTCC<br>TCCGCCTTGCCAGCCGCTGCGCCCGAGCTGGCCTGC
CRM9	*THBS1*	55	CCCTTGATGAGAATACGCACACCGCCCCCAAGCGGCCGGCCGAGGGAGCGCCGC<br>G
CRM10	*THBS1*	280	AATACAATAATAGGCAGAGAGTAATTTATTACTCTATGGGTCTGCTCTGTAAATAGC<br>TGAAGACTCTGGAGCCAGATGGTTCTGCAAATTCTCCAAACAGGAGTCACGTTAAG<br>AAGCACGAGTGGGCACAAAAACTGTTTTTCAAGACACAATTTCAATTTGGCTTGTG<br>GAAACTGGATACGAGTAAGTTTCCTTAAAATTCGAGTAGAAAGCAGCTGTCCTCCC<br>CGGGCCCCTTGATGAGAATACGCACACCGCCCCCAAGCGGCCGGCCGAGGGAG<br>CG
CRM11	*VWF*	237	ACTGTCTTGCTGTTATGTAGCCCAGGGGCTGTGGAGTCCCCACTGCTGGGGGAGA<br>TAAAGCCCAAGCTGTGACATCCACCAACCACCCTCCCTTTCCCACCACAATAGCTG<br>TGAGCTGCCACAACAGGGGATTGGCCTCCTTTTAATTACCAAAAGGAAACAATGAA<br>AGGAAATGGTATTAGAAGTACCTCCAAGCTGATAAAGCTTTGTAGAGTTTGGAGGG<br>AGACCTCCTGGCAG

Supplementary Table S3. Primer and probe sequences.

Primer Name	Primer Sequence
ApoAI F (BAEC)	5´-AGAATATGTGGCCCAGTTTGAAG-3´
ApoAI R (BAEC)	5´-TCCCAGTTGTCCAGGAGCTT-3´
ApoAI P (BAEC)	5´-56-FAM/CTCCGCCTT/ZEN/TGGAAAGCAACTCAACC/3IABkFQ-3´
ApoAI F (HAEC, HUVEC)	5´-ACCTTGGCTGTGCTCTTC-3´
ApoAI R (HAEC, HUVEC)	5´-CAGGCTGTCCCAGTTGTC-3´
ApoAI P (HAEC, HUVEC)	5´-56-FAM/CAGTCAAGG/ZEN/ACAGCGGCAGAGAAT/3IABkFQ-3´
GAPDH F (BAEC)	5´-TGACCCCTTCATTGACCTTCA-3´
GAPDH R (BAEC)	5´-GCCTTGACTGTGCCGTTGA-3´
GAPDH P (BAEC)	5´-56-FAM/TCCAGTATG/ZEN/ATTCCACCCACGGCAA/3IABkFQ-3´
GAPDH F (HAEC, HUVEC)	5´-GGTGTGAACCATGAGAAGTATGA-3´
GAPDH R (HAEC, HUVEC)	5´-GAGTCCTTCCACGATACCAAAG-3´
GAPDH P (HAEC, HUVEC)	5´-56-FAM/AGATCATCA/ZEN/GCAATGCCTCCTGCA/3IABkFQ-3´
oPRE F	5´-CAACTCCTTTCTGGGACTTTC-3´
oPRE R	5´-AGGCGGCGATGAGTTCTG-3´
oPRE P	5´-56-FAM/TTTCCCCCT/ZEN/CCCGATCGCCA/3IABkFQ-3´

Species-specific primers and probes were used for bovine aortic endothelial cells (BAEC), human aortic endothelial cells (HAEC), and human umbilical vein endothelial cells (HUVEC). ApoAI, apolipoprotein AI; F, forward; R, reverse; P, probe; GAPDH, glyceraldehyde phosphate dehydrogenase; oPRE, optimized woodchuck hepatitis virus post-transcriptional regulatory element.
